# [^68^Ga]Ga-4HMSA a promising new PET tracer for imaging inflammation

**DOI:** 10.1186/s13550-021-00856-w

**Published:** 2021-10-30

**Authors:** Shigufa Kahn Ali, Samia Ait-Mohand, Véronique Dumulon-Perreault, Brigitte Guérin

**Affiliations:** 1 Department of Nuclear Medicine and Radiobiology, Faculty of Medicine and Health Sciences, Université de Sherbrooke, Sherbrooke, QC J1H 5N4 Canada; 2 Sherbrooke Molecular Imaging Center (CIMS), CRCHUS, 3001, 12e Avenue Nord, Sherbrooke, QC J1H 5N4 Canada

**Keywords:** [^68^Ga]Ga-4HMSA, Radiotracer, Positron emission tomography (PET), Inflammation, PET imaging

## Abstract

**Background:**

Imaging diagnosis of inflammation has been challenging for many years. Inflammation imaging agents commonly used in nuclear medicine, such as [^67^Ga]Ga-citrate and 2-deoxy-2-[^18^F]fluoro-d-glucose ([^18^F]FDG) showed some limitations. The identification of a radiotracer with high specificity and low radiation dose is clinically important. With the commercialization of ^68^Ge/^68^Ga generators and the high ^68^Ga cyclotron production capacity, the study of ^68^Ga-based tracer for inflammation has increased and shown good potential. In the present work, we report the synthesis of 4HMSA, a new acyclic chelator, and its first investigation for ^68^Ga complexation and as a new positron emission tomography (PET) imaging agent of inflammation in comparison to [^68^Ga]Ga-citrate.

**Results:**

The present experimental studies have shown that the novel [^68^Ga]Ga-4HMSA is stable allowing imaging of inflammation in a preclinical model of adjuvant- and pathogen-based inflammation involving intraplantar injection of complete Freund’s adjuvant (CFA). We also found that [^68^Ga]Ga-4HMSA displayed similar uptakes in the inflamed paw than [^68^Ga]Ga-citrate, which are superior compared to those of contralateral (non-injected) paws at days 1–3 from PET imaging. [^68^Ga]Ga-citrate accumulated in the upper body of the animal such as the liver, lungs and the heart, whereas the [^68^Ga]Ga-4HMSA revealed low uptakes in the majority of the organs and was cleared relatively rapidly from blood circulation through the kidneys and bladder.

**Conclusion:**

The results highlight the potential of [^68^Ga]Ga-4HMSA as an interesting alternative to [^68^Ga]Ga-citrate for inflammation imaging by PET. The new PET tracer also offers additional advantages than [^68^Ga]Ga-citrate in term of dosimetry and lower overall background activity.

## Background

Inflammation plays a significant role in a wide range of disease processes. The identification and the localization of inflammation is critical for the adequate treatment of patients. Consequently, the development of more sensitive radiotracers for molecular imaging of inflammation by positron emission tomography (PET) may provide new insights into the detection and the treatment at early stage disease [1]. Over the past years, many radiopharmaceuticals including 2-deoxy-2-[^18^F]fluoro-d-glucose ([^18^F]FDG) and ^68^Ga-based tracers [2] have received considerable attention and some of them have shown promising results in the field of inflammation imaging [3–7].

[^67^Ga]Ga-citrate, the SPECT predecessor of [^68^Ga]Ga-citrate was a prime radiotracer for imaging inflammation of musculoskeletal origin [3] for over 40 years but several shortcomings limit its clinical application including poor image quality and resolution as well as its high energy gamma rays delaying imaging of up to 72 h. In contrast, the PET counterpart [^68^Ga]Ga-citrate has a shorter physical half-life (T_½_ = 67,71 min) and improved imaging options such as shorter injection-to-imaging time, improved dosimetry and better spatial resolution in terms of image quality [8]. With the commercialization of ^68^Ge/^68^Ga generators and the high ^68^Ga cyclotron production capacity [9–11], the study of ^68^Ga-based tracer for inflammation has increased considerably and is well justified.

**Fig. 1:**
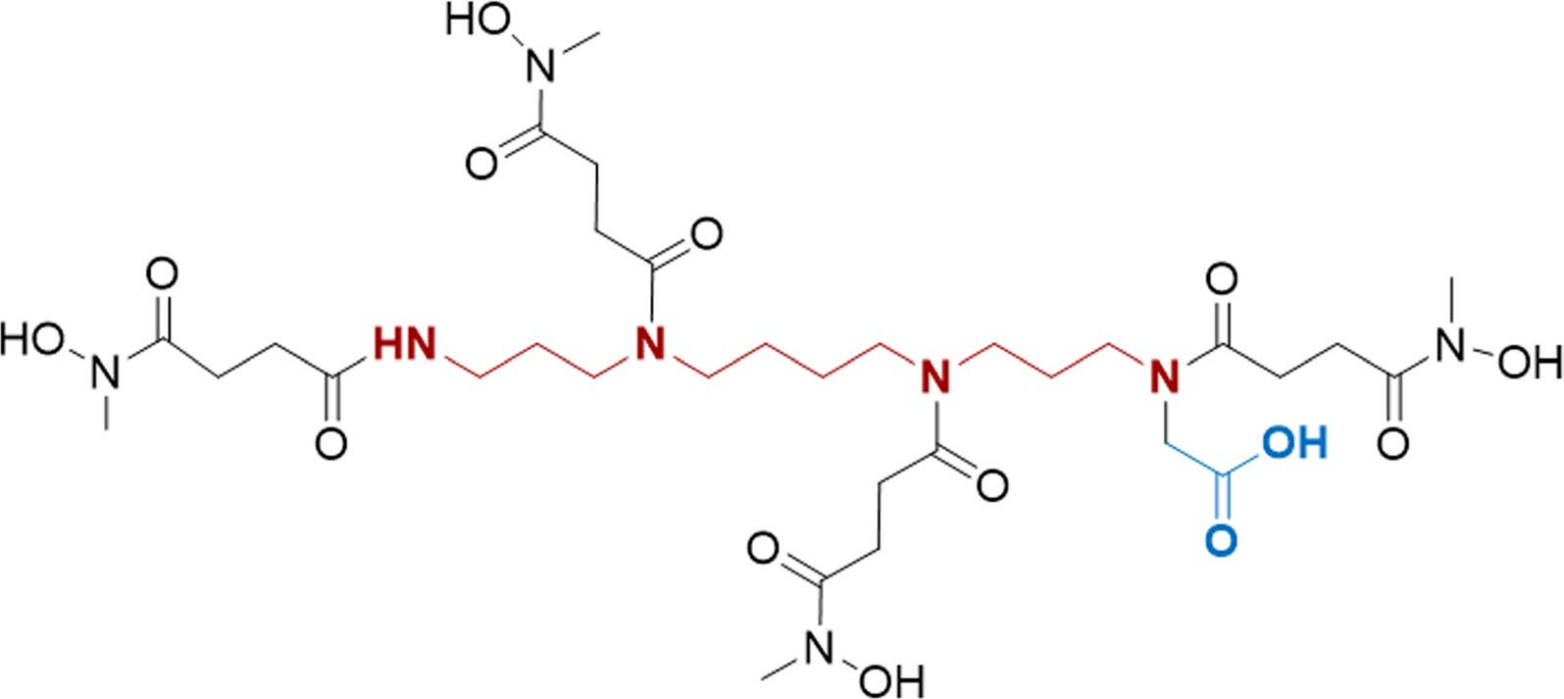
Structure of the new acyclic chelator 4HMSA

In our previous work, we reported the synthesis of *N*-methylhydroxamate derived from tetraaza- and triazamacrocycles, named DOTHA_2_ and NOTHA_2_ [12]. The promising results obtained with this new class of chelators led us to become interested in whether spermine, a natural tetraamine, could be advantageously used as a template for preparing an acyclic chelator with four *N*-hydroxy-*N*-methyl succinamide pendant arms. Effectively, the new acyclic chelator named 4HMS allowed fast and highly selective labeling with ^89^Zr revealing excellent imaging properties using PET compared to [^89^Zr]Zr-desferoxamine (DFO) analogue [13]. Therefore, we aimed to develop a bifunctional variant of the 4HMS for further evaluation and application in inflammation-based PET imaging. This new bifunctional chelator called 4HMSA is functionalized with a carboxylic acid group at one of the terminal amine in order to improve its solubility at physiologic pH (Fig. 1). Our hypothesis is that 4HMSA will act as a transporter of ^68^Ga, which will modulate favorably its biodistribution profile making the resultant [^68^Ga]Ga-4HMSA an appropriate PET tracer for inflammation imaging.

In this current study, we present the synthesis of 4HMSA as well as the detailed chemical and radiochemical investigations as a [^68^Ga]Ga-4HMSA PET tracer. In order to investigate the full potential of 4HMSA, we studied the radiolabeling performance under various chelator concentration and pH conditions in presence of [^68^Ga]GaCl_3_. The complexation properties and stability of [^68^Ga]Ga-4HMSA complex were examined and compared with that of [^68^Ga]Ga-NOTA and [^68^Ga]Ga-DOTA, which are the most widely applied chelating systems for radiolabeling of biomolecules with ^68^Ga. Apparent molar activity (AMA) was calculated and metal competition, transchelation with DTPA, apo-transferrin and protein binding studies were also investigated. We validated [^68^Ga]Ga-4HMSA as a new inflammation PET tracer and compared this new ^68^Ga- chelator to [^68^Ga]Ga-citrate in a preclinical model of adjuvant- and pathogen-based inflammation involving intraplantar injection of complete Freund’s adjuvant (CFA). The aim of this study is to demonstrate that [^68^Ga]Ga-4HMSA is as effective as [^68^Ga]Ga-citrate for PET imaging of inflammation while having a better specificity, pharmacokinetic and a lower radiation dose.

## Methods

### General

^68^Ga was obtained from a commercially available generator system (Eckert & Ziegler Isotope Products Inc.), eluted and purified according to manufacturer’s instructions. All starting compounds and reagents were used as obtained without further purification. Anhydrous solvents were obtained commercially and used only where indicated. Diluted HCl for elution of ^68^Ge/^68^Ga generator was prepared from HCl (trace metal) and high-purity water. High-purity water was also used for all radiochemical works, including the preparation of solutions of precursors and buffers. Hydrochloric acid (99.999%), apo-transferrin (≥ 98.0%), DTPA (≥ 99%), were obtained from Sigma-Aldrich (Saint-Louis, MO, USA). Iron chloride (FeCl_3_·6H_2_O, 99%), cobalt chloride (CoCl_2_ 6H_2_O, 97%) and magnesium chloride (MgCl_2_, 98%) were obtained from Fisher Scientific (Ottawa, ON, CA). Copper chloride (CuCl_2_, 99%) and nickel chloride (NiCl_2_ 6H_2_O, 98%) were obtained from Sigma-Aldrich (Saint-Louis, MO, USA). Acetonitrile (CH_3_CN) (HPLC grade, 99.9%) and High-purity water (Optima LC/MS, ultra-high-performance liquid chromatography ultraviolet grade, 0.03 mm filtered) were purchased from Fisher Scientific (Ottawa, ON, CA). ^1^H and ^13^C NMR spectra were recorded in deuterated solvents on a Brucker Ascend 400 NMR instrument. The residual solvent peaks have been used as internal references. The peak multiplicities are described as follows: s (singlet), d (doublet), t (triplet), q (quartet), quin (quintet), m (multiplet), and br (broad). High-resolution mass spectrum (HRMS) was recorded on a Triple TOF 5600, ABSciex mass spectrometer. Analytical HPLC was performed on an Agilent 1200 system (Agilent Technologies, Mississauga, Ontario, Canada) equipped with a Zorbax Eclipse XDB C18 reversed-phase column (4.6 × 250 mm, 5 μ) and Agilent 1200 series diode array UV–vis (Agilent Technologies) using a linear gradient of CH_3_CN (0.05% TFA) into H_2_O (0.025% TFA) (0–76%) over 23 min followed by CH_3_CN into H_2_O (76–100%) over 1 min, and CH_3_CN into H_2_O (100–0%) over 6 min with a flow rate of 1 mL/min. UPLC chromatograms were obtained from Waters UPLC H-Class equipped with an ELSD detector and a γ-counter from Eckert-Zeigler (Washington D.C. U.S.A.). The column used was a Waters BEH C18 column (2.1 × 50 mm) 1.7 µm and eluent was a gradient of CH_3_CN (0.05% AcOH)/H_2_O (0.05% AcOH). Alburex-25 (Human plasma, USP) was supplied by Grifols Canada Ltd. (formerly Talecris), CSL Behring. Instant thin-layer chromatography (ITLC-SG) was acquired from Agilent Technology (Santa Clara, CA). All glassware was cleaned with chromic sulfuric acid (Fisher Scientific). The radio-TLC were scanned using an Instant Imager scanner (Bioscan, DC, U.S.A.). Benzenesulfonic resin (CUBCX123) was bought from UCT, Inc (Bristol, PA, USA). Radioactivity measurements were performed in an ionization chamber (CRC-25PET; Capintec) on the ^68^Ga setting to control process efficiency. The labeling efficiency of [^68^Ga]Ga-4HMSA, [^68^Ga]Ga-NOTA and [^68^Ga]Ga-DOTA was assessed using ITLC-SG with 0.1 M sodium citrate (pH = 5) as eluant.

### Preparation of [^68^Ga]Ga-citrate

The ^68^Ge/^68^Ga generator was eluted with 0.1 M HCl and the radioactivity was transferred to a cation exchange cartridge (CUBCX123). The cartridge was rinsed with 5 mL of physiological saline to remove potential ^68^Ge residues. ^68^Ga was eluted from the cartridge with 2 mL of sodium citrate solution (68 mM, pH 5.5) and then diluted with 3 mL of saline solution in the product vial. The radiochemical purity of [^68^Ga]Ga-citrate solution was evaluated in two solvent mixtures and two stationary phases (ITLC-SG and Whatman paper n^o^2) and compared with the retention time of the [^68^Ga]GaCl_3_.

### Preparation of (*N*1, *N*4, *N*9-tri-*tert*-butoxycarbonyl)-1,12-diamino-4,9-diazadodecane 3

The tri-Boc-protected derivative was prepared according to the literature [14] with slight modifications. Spermine (4.0 g, 20 mmol) was dissolved in 30 mL of CH_2_Cl_2_ at 0 °C. Ethyl trifluoroacetate (2.4 mL, 20 mmol) in 5 mL of CH_2_Cl_2_ was added dropwise and the mixture was then left stirring for 1 h at room temperature. After evaporation of the solvent, the crude residue was taken up in 60 mL of THF and NEt_3_ (14.0 mL, 60 mmol) was added. A solution of (Boc)_2_O (17.5 g, 60 mmol) in 20 mL of THF was added and the mixture was stirred for 3 h. After evaporation of the solvent, the residue obtained was taken up with 100 mL of 8/2 MeOH/H_2_O mixture and Cs_2_CO_3_ (20.0 g, 62 mmol) was added and the whole mixture was heated to reflux for 3 h. The solvents were evaporated, and the residue treated with diisopropylether and aqueous solution of HCl (0.5 M). The oily insoluble organic material in both aqueous and organic solvents was decanted from a three-layer system. After drying, the compound was obtained as a pale-yellow oil. The product **3** was used without further purification. Yellow oil. (50%). ^1^H NMR (400 MHz, CDCl_3_): *δ* = 1.33 (s, 27H), 1.38–1.34 (m, 4H), 1.55–1.52 (m, 2H), 1.84–1.75 (m, 2H), 2.81 (t, J = 7.3 Hz, 2H), 3.00–2.96 (m, 2H), 3.18–3.07 (m, 6H), 3.28–3.25 (m, 2H). ^13^C NMR (100 MHz, CDCl_3_): *δ* = 156.0, 99.7, 28.6, 28.21, 25.5.

### Preparation of benzyl (9,14-bis(***tert***-butoxycarbonyl)-2,2-dimethyl-4-oxo-3-oxa-5,9,14-triazaheptadecan-17-yl) glycinate 4 [15]

A solution of tri-Boc-spermine **3** (6.0 g, 10 mmol), benzyl bromoacetate (1.9 mL, 12 mmol) and TEA (2.6 mL, 16.7 mmol) in THF (120 mL) was stirred at room temperature overnight. The precipitate was filtered off and the filtrate was concentrated under reduced pressure. The crude product was purified by silica gel chromatography (100% diethylether, then 10% MeOH in CH_2_Cl_2_) to give **4** (5.3 g, 72%) as a pale yellow coloured viscous liquid. ^1^H NMR (400 MHz, CDCl_3_): *δ* 1.44–1.48 (m, 31H), 1.64–1.73 (m, 4H), 2.72 (t, *J* = 7.0, 2H), 3.10–3.26 (m, 10H), 3.44 (s, 2H), 5.16 (s, 2H), 7.32–7.37 (m, 5H). *ESI MS* (LC–ESI–MS) *m*/*z*: calcd, (650.84); found, 651.51 (M + 1).

### Preparation of benzyl (3-((4-((3-aminopropyl)amino)butyl)amino)propyl) glycinate sodium salt. 5

A solution of 4 M HCl in dioxane (50 mL) was added to a stirring solution of **4** (3.0 g, 4.6 mmol) in CH_2_Cl_2_ (10 mL) at 25 °C under nitrogen. After 2 h, the solution was concentrated in vacuo and co-evaporated with toluene (3 × 10 mL) to yield quantitatively compound **5** as a poly-HCl white salt. ^1^H NMR (400 MHz, D_2_O): *δ* 1.62 (t, J = 7.2 Hz, 2H), 2.15–1.95 (m, 4H), 3.12–2.90 (m, 12H), 3.95 (s, 2H), 5.12 (s, 2H), 7.22–7.37 (m, 5H). ^13^C NMR (100 MHz, D_2_O): *δ* = 166.85, 134.51, 129.02, 128.89, 128.76, 128.62, 71.55, 70.71, 68.54, 60.27, 57.41, 47.42, 47.00, 44.52, 44.43, 43.21, 36.52, 23.72, 22.74, 22.49, 16.78. *ESI MS* (LC–ESI–MS) *m*/*z*: calcd, (350.51); found, 351.29 (M + 1).

### Preparation of benzyl 12,17,21-tris(4-((benzyloxy)(methyl)amino)-4-oxobutanoyl)-3-methyl-4,7-dioxo-1-phenyl-2-oxa-3,8,12,17,21-pentaazatricosan-23-oate 6

To a solution of **5** (0.5 g, 1.4 mmol) in DMF (5 mL) was added DIEA (1 mL, 5.7 mmol) and the mixture was left stirring for 1 h at room temperature. *N*-methyl-*N* (benzyloxy) succinimide (1.4 g, 5.7 mmol), DIEA (2 mL, 11.4 mmol) and HATU (2.2 g, 5.7 mmol) were stirred in anhydrous DMF (10 mL) for 1 h. This solution was added at 0 °C to the first suspension and the reaction mixture was left stirring to room temperature for 24 h. The reaction mixture was washed with 10% NaHCO_3_ solution followed by water. The organic phase was dried over anhydrous Na_2_SO_4_ and was then removed with a rotary evaporator. The crude product was purified by Biotage flash chromatography to yield 83% of protected 4HMSA **6** as a fluffy beige solid after lyophilisation. The product purity was confirmed to be 100% by analytical reversed phase HPLC: retention time of 24.1 min. ^1^H NMR (400 MHz, CDCl_3_): *δ* 1.52–1.85 (m, 8H), 2.40–2.85 (m, 16H), 3.20–3.45 (m, 24H), 4.12–4.25 (m, 4H), 4.85–4.95 (m, 8H), 5.2 (M, 2H), 7.45–7.55(m, 24H), 8.50–7.50 (s broad, 2H). ^13^C NMR (100 MHz, CDCl_3_): (mixture of rotamers), 174.12, 172.95, 172.93, 172.61, 172.57, 172.44, 172.39, 171.85, 169.44, 169.41, 169.32, 135.56, 135.52, 135.43, 135.11, 135.02, 134.61, 129.27, 129.08, 128.87, 128.68, 128.59, 128.56, 128.47, 128.45, 128.41, 128.38, 76.46, 67.45, 67.33, 66.94, 65.85, 50.83, 48.29, 47.52, 47.02, 46.70, 45.32, 45.19, 44.83, 43.51, 42.78, 42.60, 36.92, 36.21, 33.66, 30.58, 27.62, 27.50, 27.48, 27.35, 27.28, 27.24, 27.10, 27.04, 26.32, 26.17, 26.13, 26.09, 25.90, 25.73, 25.05, 25.00, 24.92. *ESI MS* (LC–ESI–MS) *m*/*z*: calcd, (1227.5); found, 1228.6 (M + 1), 615.4 (M/2 + 1).

### Preparation of 2-hydroxy-11,16,20-tris(4-(hydroxy(methyl)amino)-4-oxobutanoyl)-3,6-dioxo-2,7,11,16,20-pentaazadocosan-22-oic acid 7

A solution of protected 4HMSA **6** and 10% Pd/C (20% w/w) was suspended in methanol. The reaction mixture was purged with hydrogen gas at room temperature overnight. The crude was filtered over celite and washed with methanol. The solvent was evaporated co-evaporated with diethyether to give quantitatively 4HMSA **7** as a colorless solid. The compound is very hygroscopic and must be kept under nitrogen at − 20 °C. The HPLC chromatogram revealed a mixture of three conformational isomers or conformers in variable compositions. The product purity was confirmed to be 100% with a retention time of 11.0 min. ^1^H NMR (400 MHz, DMSO): *δ* 1.42–1.75 (m, 8H), 2.35–2.75 (m, 18H), 2.85–3.38 (m, 24H), 3.85 (m, 1H), 4.17 (m, 1H) 7.71–7.92 (m, 1H), 9.80 (s broad, 3H). ^13^C NMR (100 MHz, DMSO): (mixture of rotamers), 172.85, 172.73, 172.67, 172.01, 171.90, 171.89, 171.85, 171.59, 171.48, 171.43, 171.38, 49.06, 47.86, 45.18, 45.16, 44.86, 44.85, 37.84, 36.81, 36.60, 36.26, 36.18, 30.21, 30.26, 29.03, 29.02, 28.09, 28.07, 27.78, 27.71, 27.52, 27.48, 27.44, 27.26, 27.22, 27.19, 27.10, 27.06, 26.28, 25.21, 25.19, 25.11. LRMS (LC–ESI–MS) *m*/*z*: calcd, (777.8); found, 778.4 (M + 1), 390.0 (M/2 + 1).

### Radiolabeling of 4HMSA 7

^68^Ga was eluted from a ^68^Ge/^68^Ga generator using 0.1 M HCl. The ^68^Ga elution was pre-purified and concentrated on a CUBCX123 column. The purified ^68^Ga^3+^ was obtained by eluting the column with a mixture of 12.5 μL of 5.5 M HCl and 500 μL of 5 M NaCl. To this eluate was slowly added a solution of 100–150 μL ammonium acetate buffer 1 M, pH = 4.5 and mixed by brief shaking, resulting in a pH ranging from 3.5–3.8. Varying amounts (0.03–10 nmol) of 4HMSA dissolved in water were mixed with 37–40 MBq of neutralized [^68^Ga]GaCl_3_ as described above. The labeling mixtures were allowed to react for 5–10 min at room temperature. The radiolabeling yield for [^68^Ga]Ga-4HMSA was confirmed by radio-TLC using 0.1 M sodium citrate (pH = 5) as a mobile phase. In this system, free ^68^Ga eluted with the solvent front while [^68^Ga]Ga-4HMSA complex remained at the origin.

### Determination of apparent molar activity (AMA)

AMA (GBq/μmol) of [^68^Ga]Ga-4HMSA was calculated in triplicate by titration of the chelator with purified [^68^Ga]GaCl_3_. Solutions of 4HMSA (150 μL in 4 mL polypropylene tube) at different concentrations (1–12 × 10^−2^ nmol) were prepared via serial dilutions. [^68^Ga]GaCl_3_ solution was adjusted to a final pH of 3.5–3.8 with NH_4_OAc buffer (1 M pH = 4.5) and added (22 μL, ~ 15 MBq) to each tube to give a total volume of 250 μL. In addition, a blank tube with ~ 15 MBq of [^68^Ga]GaCl_3_ in 250 μL of water was prepared. After mixing and incubation at RT for 10 min, the AMA was determined by measuring [^68^Ga]Ga-4HMSA labeling efficiency in each tube by TLC using 0.1 M sodium citrate (pH = 5) as mobile phase. TLC plates were analyzed using a radio-TLC scanner. The percentages of complexation were plotted as a function of amount of chelator (nmol) and the AMA was determined when > 95% complexation was obtained.

### Comparative ^68^Ga labeling of 4HMSA, DOTA, and NOTA

#### Concentration effect

A series of reactions were performed in which a solution of [^68^Ga]GaCl_3_ (approx. 25 MBq) was added to a solution of each chelator at a concentration in the range of 1 mM to 100 nM. The final pH of the reaction solution was 3.5–3.8 (using NH_4_OAc buffer 1 M pH = 4.5). A side-by-side comparison was made of the ^68^Ga-chelating efficiency of 4HMSA with NOTA and DOTA using instant thin layer chromatography (ITLC) at progressively lower chelator concentration (Fig. 4).

#### pH effect

A series of reactions were undertaken in which a solution of [^68^Ga]GaCl_3_ (approx. 25 MBq) was added to a solution of each chelator at 10 μM concentration. The final pH of the reaction solution was adjusted at 1–2, 2–3 and 3.5–3.8 using NH_4_OAc buffer 1 M pH = 4.5. Labeling of 4HMSA and NOTA were performed at room temperature and DOTA was labeled at 80 °C as a function of time (Fig. 5).

### Apo-transferrin challenge studies

Apo-transferrin (77 kDa) was diluted with 10 mM of sodium carbonate (pH 6.5–7) to reach a final concentration of 10 mg/mL. Apo-transferrin (10 equiv) (820 μL, ∼1.3 × 10^5^ nM) was added to diluted [^68^Ga]Ga-4HMSA (820 μL, ∼40 MBq). The solution was divided into four aliquots that were incubated at 37 °C for 30, 60 and 120 min. An aliquot was transferred to Amicon Ultra 0.5 mL 50 kDa filter (Merck KGaA, Darmstadt, Germany) and centrifuged at 6600 rpm for 12 min. In this system, [^68^Ga]Ga-4HMSA passed through the filter, while apo-transferrin and [^68^Ga]Ga-apo-transferrin remained on the filter. The radioactivity in each fraction was measured using a dose calibrator. The percentage of transchelation was then calculated. The study was performed in triplicate.

### Transchelation studies using DTPA

4HMSA (100 μL, 100 μM) was labeled [^68^Ga]GaCl_3_ (15 MBq) at room temperature for 10 min and then incubated with 50 and 1000-fold excess of DTPA (200 μL of 5 and 50 mM), respectively, at 37 °C and pH = 7.2 for a period of 80 min. To maintain the pH, 100 μL of sodium acetate buffer (0.5 M, pH ≈ 5.5) was added to the solutions. Each solution was prepared in triplicate, and the quality control was performed, as described above for different time points.

### Transmetallation challenge studies

4HMSA (1 mL, 200 μM) was labeled with 100 MBq of neutralized [^68^Ga]GaCl_3_ diluted in 1 mL of water at room temperature for 10 min for a total volume of 2 mL. The chelator was then incubated with tenfold excess of various metal cations [iron(III) chloride, cobalt(II) chloride, copper(II) chloride, magnesium(II) chloride and nickel (II) chloride] at pH = 2.0 and 7.4 (Table 3). Each metal solution (200 μL) was added to 200 μL of [^68^Ga]Ga-4HMSA, and the solutions were incubated at 37 °C. To maintain the pH, sodium acetate buffer (0.1 M, pH ≈ 5.5) was added to the solutions. The samples were monitored for 60 min by radio-TLC to determine the percentage of the intact [^68^Ga]Ga-4HMSA. All the studies were done in triplicate.

### Plasma stability studies

Plasma stability studies were realized as previously described by our group [12]. Briefly, the study was carried out by incubating [^68^Ga]Ga-4HMSA (60 MBq in 200 µL PBS buffer) in 200 μL of human plasma and the solution was incubated at 37 °C for up to 2 h. The plasma was then mixed twice with (1:1) acetonitrile to precipitate all the proteins. The samples were subjected to vortex mixing for 1 min and then centrifugation for 15 min at 6600 rpm. Plasma proteins were separated from the soluble component by ultracentifugation and the radioactivity was counted. The supernatant fraction was assayed by radio-iTLC on C18 plates; free [^68^Ga]GaCl_3_ and [^68^Ga]Ga-4HMSA were used as standards. The radio-TLCs were eluted with 0.1 M sodium citrate buffer at pH 5.5 using an Instant Imager system (Bioscan, DC, U.S.A.) for the radiodetection.

### Protein binding in rodent plasma

[^68^Ga]Ga-4HMSA (5 nmol / 250 MBq) was incubated in fresh rat plasma (250 μL) and in PBS (250 μL) (control) at 37 °C for various time points (15, 30, 45, 60, and 120 min). The plasma was then mixed twice with (1:1) acetonitrile to precipitate all the proteins. The samples were subjected to vortex mixing for 1 min and then centrifugation for 15 min at 6600 rpm. Plasma proteins were separated from the soluble component by ultracentifugation and the radioactivity was counted.

### Animal studies

Animal experiments were performed in adult female BALB/c mice (Charles River Laboratories, Saint-Constant, QC, Canada). The animals were maintained in animal facility, under specific pathogen-free conditions. Housing and all procedures involving animals were performed in accordance with the guidelines of the Canadian Council on Animal Care (CCAC) and were approved by the Ethics Committee of the Université de Sherbrooke.

### Induction of inflammation

Mice were injected subcutaneously in the left footpad with 10 μL of complete Freud adjuvant (CFA) solution (0.5 mg/mL), which contains components of Mycobacterium tuberculosis (M. tuberculosis). The solution was made with a water-in-oil emulsion using the heat-killed mycobacteria in mineral oil with saline in a ratio of 1:1. The inflammation was studied at 1, 2, 3- and 7-days post-CFA administration.

### PET Imaging studies

The animals were anaesthetized by inhalation of isoflurane (induction 2–2.5%, maintained 1–1.5% oxygen flow 1–1.5 L/min) during *i.v.* injection and PET imaging procedures. A catheter was installed in the caudal vein for the administration of the radiotracer. The mouse was positioned in the field of view of the PET/CT scanner (Triumph/LabPET8™ platform (Gamma Medica, Northridge, CA), and a 45 min dynamic acquisition was acquired followed immediately by the administration of either [^68^Ga]Ga-4HMSA or [^68^Ga]Ga-citrate (∼ 9 MBq, 0.2 mL). The dynamic acquisition was followed by 2 min CT scan. Static PET/CT images were acquired between 45 and 60 min after injection of [^68^Ga]Ga-citrate for these mice groups: 2, 3- and 7-days post-inflammation. The images were reconstructed using the three-dimensional maximum likelihood estimation method algorithm, and analysis was performed using AMIDE software [16]. To quantify the radiotracer uptake, regions of interest (ROIs) were drawn around organs. The ROI activity was expressed as percent of injected dose per gram of tissue (% ID/g).

### Biodistribution studies

Under isoflurane anesthesia (induction 2–2.5%, maintained 1–1.5% oxygen flow 1–1.5 L/min) the mice were injected via the caudal vein with either [^68^Ga]Ga-4HMSA or [^68^Ga]Ga-citrate (∼9 MBq, 0.2 mL). At 60 min post injection, the mice were euthanized by CO_2_ inhalation under isoflurane anesthesia and the organs of interest were collected, washed with PBS, blotted dry, weighted, and counted in a γ-counter (HIDEX AMG, Gamble Technologies Limited, Mississauga, Canada). The results were expressed in terms of percentage of injected dose per gram (% ID/g).

### Statistical analysis

All statistical analyses were performed using Prism 7.03 for Window (GraphPad software, Inc., La Jolla, CA, USA). All results are reported as mean ± SD. The number of animals ranged from 2 to 8. Differences were considered statistically significant at *p* ≤ 0.05 (paired *t-test*).

## Results

[^68^Ga]Ga-citrate solution was prepared as described by Aghanejad et al*. *[17]*.* For the quality control of [^68^Ga]Ga-citrate, the best eluant system was considered to be a mixture of sodium acetate/ acetic acid/H_2_O in both ITLC and Whatman paper n^o^ 2 in the stationary phase. Under these conditions, the R_f_ of [^68^Ga]Ga-citrate was 0 compared to 0.9 for [^68^Ga]GaCl_3_.

**Scheme 1:**
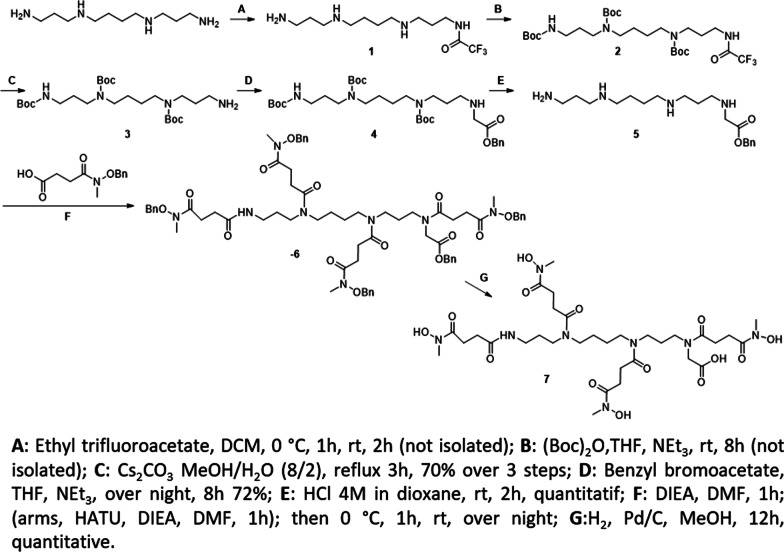
Synthesis of 4HMSA **7**

In order to achieve the synthesis of 4HMSA (compound **7**, Scheme 1) in high yield, we adopted a strategy inspired by the synthesis of *p*-SCN-Bn-HOPO [14] starting from a spermine core and *N*-methyl-*N* (benzyloxy) succinimide previously described [13]. The synthesis consists in selective protection on one of the primary amines of spermine with a trifluoro-acetyl moiety. The ratio of primary amine toward the protecting reagent, ethyl trifluoroacetate is critical in order to afford the mono-trifluoroacetamide **1**. In one pot, the resulting free amines of **1** were protected with (Boc)_2_O to give the fully protected spermine **2**. Then, selective deprotection of the trifluoroacetamide was performed in the presence of an excess of cesium carbonate (Cs_2_CO_3_) in a mixture of methanol and water to give after purification by flash chromatography the triBoc spermine **3** in 70% overall yield. This tri-protected polyamine was converted to its benzylglycine derivative **4** by reaction with one equivalent of benzyl bromoacetate followed by deprotection with HCl 4M in dioxane to yield compound **5** quantitatively as a hydrochloride salt. This compound was used as the building block for the synthesis of 4HMSA. After optimization, the best condition for incorporation of the *N*-methyl-*N* (benzyloxy) succinimide spacer previously described [13] was the coupling with HATU in DMF. It is worth to mention that the activated intermediate was added to **5** at 0 °C and then warmed to room temperature and stirred for 24 h to afford 83% of the protected 4HMSA as a colorless solid. The last step was a catalytic hydrogenation over Pd/C affording the new chelator 4HMSA **7** in a quantitative yield. The compound **7** was characterized by HPLC, UPLC NMR, and *ESI MS*. The high degree of flexibility of 4HMSA **7** resulted in a ^13^C NMR with many overlapping multiplets due to different conformers of the ligand [14, 18, 19]. These conformers were identified as potential rotamers and not impurities since a purity greater than 99% was confirmed by quantitative HPLC analysis.

**Fig. 2:**
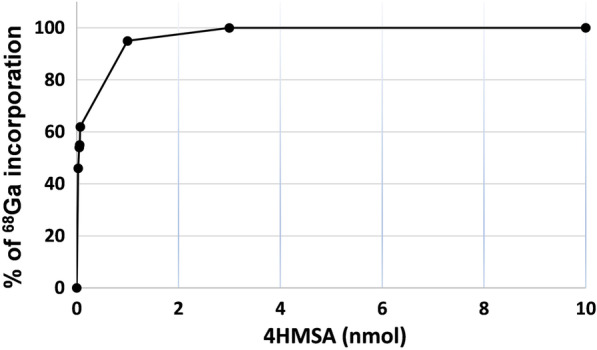
Percentage of ^68^Ga incorporation as a function of the 4HMSA concentration (nmol)

**Fig. 3:**
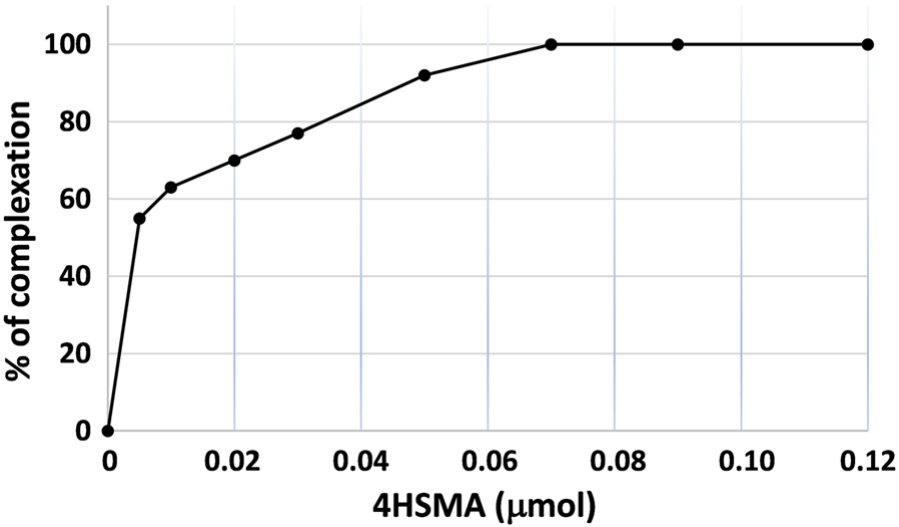
AMA (GBq/μmol) determination of ^68^Ga-4HMSA AMA calculated via titration of the chelator with purified ^68^GaCl_3_

**Fig. 4:**
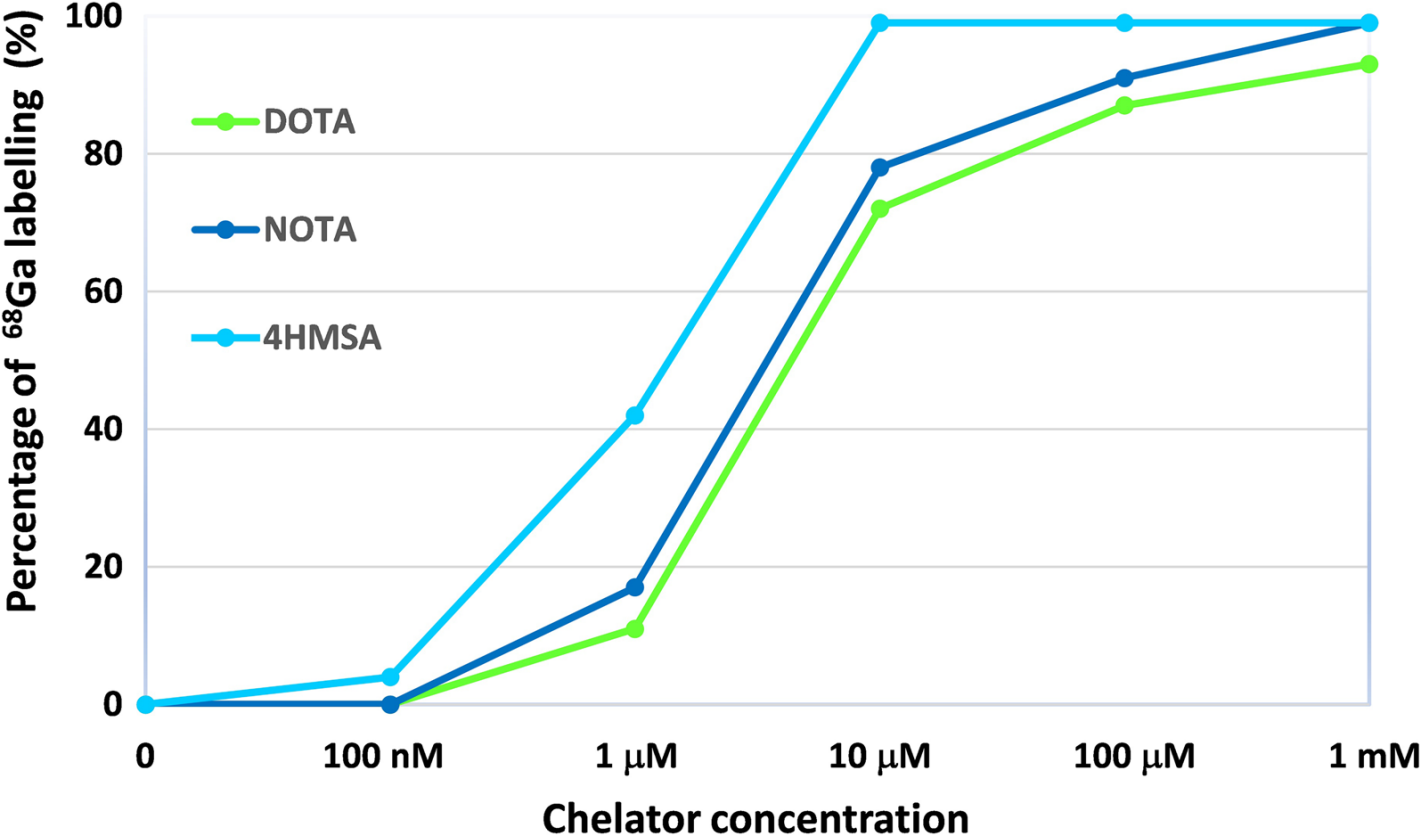
Percentage of ^68^Ga labeling with 4HMSA, NOTA and DOTA as a function of chelator concentration. A complete complexation of ^68^Ga-4HMSA was observed at a concentration of 10 μM after 5 min incubation at room temperature, while high RCYs were achieved at concentration of 1 mM after 15 min at room temperature for ^68^Ga-NOTA and 30 min at 80 °C for ^68^Ga-DOTA

A radiochemical yield (RCY) of 98–100% was achieved following the incubation 2 to 3 nmol of 4HMSA in presence of the purified [^68^Ga]GaCl_3_ (Fig. 2). AMA of [^68^Ga]Ga-4HMSA was calculated by performing a titration experiment with 4HMSA and [^68^Ga]GaCl_3_ to give 300 ± 7 GBq/μmol (Fig. 3), which was approximately eightfold higher than the value calculated for [^68^Ga]Ga-DOTA (34 ± 0.18 GBq/μmol) [9]. We then attempted a comparative ^68^Ga complexation study between 4HMSA, NOTA and DOTA, the last two being used as current “gold standard” bifunctional ^68^Ga-chelator. Here, we report the effect of the chelator concentration and the pH on the kinetic of complexation. Whilst many chelators can chelate [^68^Ga]GaCl_3_ quantitatively at high concentration, only the most efficient will continue to do so when chelator concentrations decrease. High 98–100% RCY of [^68^Ga]Ga-4HMSA was achieved for the chelator at a concentration of 10 μM after 5 min incubation at room temperature (Fig. 4), while the full complexation of [^68^Ga]Ga-NOTA and [^68^Ga]Ga-DOTA was achieved at chelator concentration of 1 mM only after 15 min at room temperature for NOTA and 30 min at 80 °C for DOTA. These interesting data demonstrated the superior labeling efficiency of 4HMSA comparing to NOTA and DOTA.

**Fig. 5:**
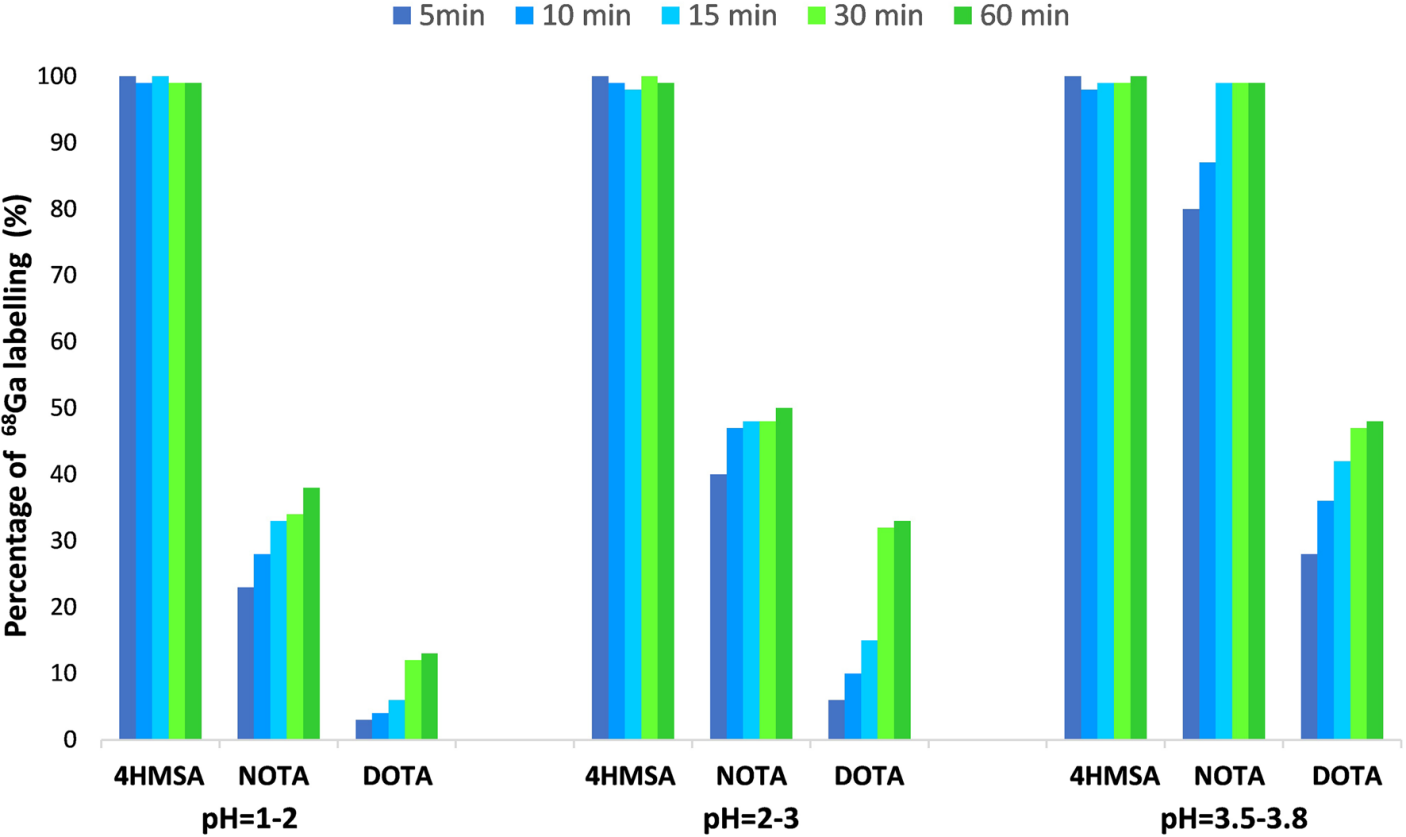
RCY of ^68^Ga with 4HMSA DOTA, NOTA under different pH conditions and as a function of time. The labeling were achieved at room temperature for 4HSMA and NOTA but at 80 °C for DOTA

Labeling efficiency of [^68^Ga]Ga-4HMSA, [^68^Ga]Ga-NOTA and [^68^Ga]Ga-DOTA was measured as a function of time and at different pH. It is worth to mention that the labeling were achieved at room temperature for 4HSMA and NOTA but at 80 °C for DOTA. The ^68^Ga radiolabeling was found to be pH dependent with DOTA and NOTA but there was no remarkable difference between studies undertaken at different pH with 4HMSA. A quantitative yield was observed only 5 min after incubation for 4HSMA even at lower pH 1–2 (Fig. 5). Low RCY were observed for DOTA and NOTA under these labeling conditions. Unreproducible and low yields were observed when the ^68^Ga-labeling of 4HMSA was done at pH > 4. A potential explanation is that ^68^Ga-labeling competes with formation of insoluble ^68^Ga-colloid that may occur at pH > 4.The results indicate that the optimal pH for radiolabeling was within a range of 3.5–3.8 for the three chelators. Under all conditions, 4HMSA “competed” most effectively for ^68^Ga^3+^ in comparison with the other chelators.

[^68^Ga]Ga-4HMSA complex should be sufficiently stable in circulation over the period of time required for imaging (~ 1 h) to resist to transchelation by competing endogenous proteins, such as apo-transferrin, and other ligands that compete for Ga^3+^ in vivo. Transchelation of [^68^Ga]Ga-4HMSA by transferrin would result in a loss of specificity and an accumulation of ^68^Ga in the liver, lungs and bone. To provide a significant test of resistance to transchelation, [^68^Ga]Ga-4HMSA was incubated with an excess of apo-transferrin for 2 h at room temperature and was reasonably stable to transferrin, with less than 10% of ^68^Ga being lost (Table 1).

**Table 1:** Transchelation of [^68^Ga]Ga-4HMSA with Apo-transferrin

Time (min)	% Apo-transferrin binding
30	6.0 ± 0.4
60	7.0 ± 0.4
120	8.0 ± 0.1

^68^Ga-4HMSA was incubated with 50 to 1000 fold excess of EDTA in PBS (pH 7.2) at 37 °C and analyzed at 15, 30 and 80 min of incubation in order to examine its susceptibility to transchelation (Table 2). Each aliquot was analyzed by ITLC using aqueous 0.1 M sodium citrate as eluant. [^68^Ga]Ga-4HMSA has shown a remarkable resistance to transchelation, remaining > 99% intact over the time until 300 fold-excess of DTPA. In contrast, the release of ^68^Ga from [^68^Ga]Ga-4HMSA was observed for higher concentration (> 500 fold-excess).

**Table 2:** Transchelation of [^68^Ga]Ga-4HMSA with DTPA

DTPA (equiv)	% of intact ^68^Ga-4HMSA		
	15 min	30 min	80 min
50	100	–	–
100	100	–	–
200	100	100	100
300	100	100	100
500	100	100	74
1000	100	77	59

A metal competition study was performed with [^68^Ga]Ga-4HMSA to see if other metal cations could outcompete ^68^Ga for the 4HMSA binding pocket. 4HMSA was labeled with ^68^Ga and then incubated with a tenfold excess of various metal salts at pH 2 and 7.4 at 37 °C (Table 3). The samples were monitored for 60 min by radio-TLC to determine the percentage of intact ^68^Ga complex. [^68^Ga]Ga-4HMSA proved to be stable (> 95% intact) when challenged with the majority of competing metals with the notable exception of Fe^3+^. In this case, after 30 min of incubation, [^68^Ga]Ga-4HMSA released ∼30% and 14% of the ^68^Ga at pH 2 and 7.4, respectively. After 1 h, the transmetalation increased to 65% and 28% at pH 2 and 7.4, respectively, indicating that the ^68^Ga-complex is more stable at physiological pH. Since 4HMSA has a structure similar to that of a siderophore, its high affinity for iron is unsurprising.

**Table 3:** Transmetallation of [^68^Ga]Ga-4HMSA in presence of various biologically relevant metal salts

Metal (10 equiv)	% of Remaining Ga^68^ complexation					
	15 min		30 min		60 min	
CoCl_2_	100^a^	100^b^	100^a^	100^b^	100^a^	100^b^
CuCl_2_	100^a^	100^b^	100^a^	100^b^	78^a^	95^b^
FeCl_3_	100^a^	100^b^	71^a^	86^b^	35^a^	72^b^
NiCl_2_	100^a^	100^b^	100^a^	100^b^	100^a^	100^b^
MgCl_2_	100^a^	100^b^	100^a^	100^b^	100^a^	100^b^

^a^pH = 2.0; ^b^pH = 7.4

**Fig. 6:**
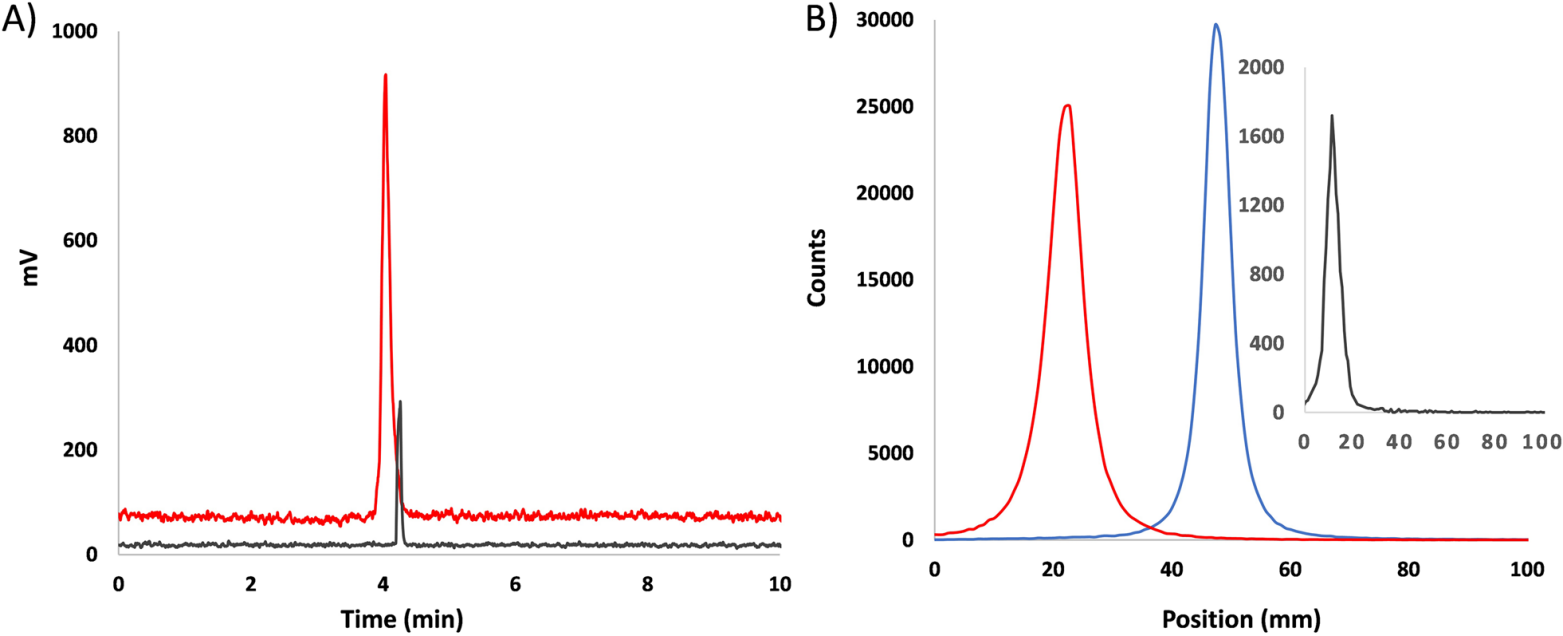
Representative radio-UPLC and iTLC profiles of **A** pure [^68^Ga]Ga-4HMSA (red line) and [^68^Ga]Ga-4HMSA in the supernatant fraction after 2 h incubation in human plasma (grey line); **B** [^68^Ga]GaCl_3_ (blue line), pure [^68^Ga]Ga-4HMSA (red line) and in the insert, [^68^Ga]Ga-4HMSA in the supernatant fraction after 1 h in rodent (grey line)

The stability of [^68^Ga]Ga-4HMSA was extensively challenged in human plasma proteins, in the presence of excess of apo-transferrin, DTPA and biologically relevant metal ions. Plasma stability studies were carried out to evaluate the in vitro and in vivo stability of [^68^Ga]Ga-4HMSA. In fact, this tracer exhibited high stability after 2 h incubation in human serum at 37 °C (Fig. 6A). To further test the stability of [^68^Ga]Ga-4HMSA, in vivo metabolism tests in rodent were performed. At 60 min post-injection of [^68^Ga]Ga-4HMSA, less than 1% dissociated ^68^Ga ions was observed in blood (Fig. 6B), suggesting substantial in vivo inertness in circulation. Protein binding studies performed by incubating [^68^Ga]Ga-4HMSA in fresh rodent plasma showed that the ability of [^68^Ga]Ga-4HMSA to bind to plasma proteins is very low since the values did not exceed ~ 10% even after 2 h (Table 4).

**Table 4:** Protein binding of [^68^Ga]Ga-4HMSA in rodent plasma

Time (min)	% protein binding
15	0.49 ± 0.06
30	0.84 ± 0.05
45	4.33 ± 0.15
60	8.75 ± 0.30
120	10.33 ± 0.21

In vivo uptake of both [^68^Ga]Ga-4HMSA and [^68^Ga]Ga-citrate was studied in a CFA mice model that induces, within days, a strong and long-lasting inflammatory reaction [20]. In mice, the footpad is an injection site well-recognized for its effectiveness in terms of producing a strong immune response [21]. Cytokines induced in the early phases following exposure to CFA are TNF-α, IL-12, IL-6, IFN-γ and several chemokines. Inflammation was studied from day 1 to day 7 post-injection.

**Fig. 7:**
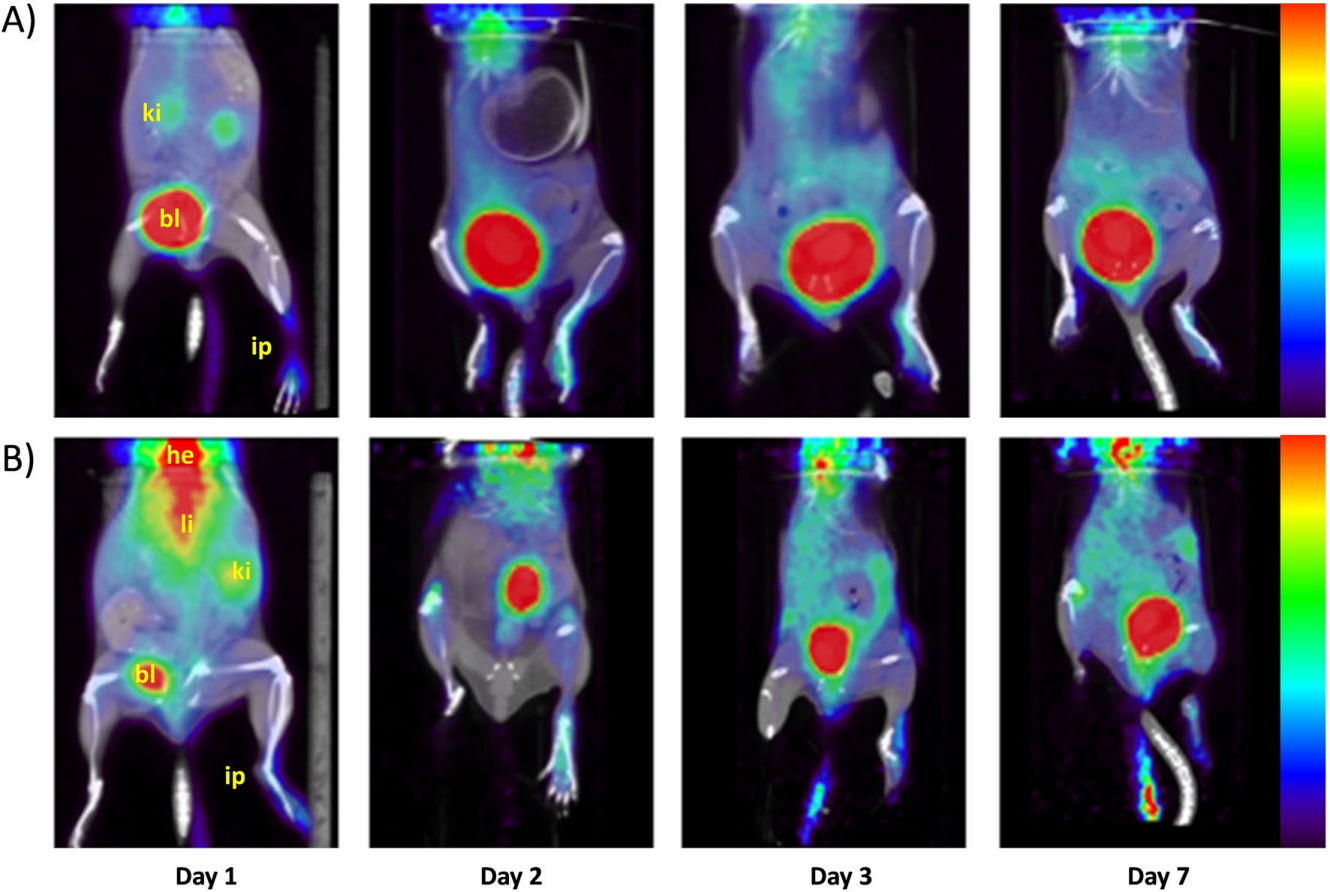
PET/CT scan of BALB/c mice injected with **A** [^68^Ga]Ga-4HMSA and **B** [^68^Ga]Ga-citrate with the left paw inflamed at days 1, 2, 3 and 7 post-CFA administration and at 15 and 45 min post-tracer injection, respectively. [^68^Ga]Ga-4HMSA and [^68^Ga]Ga-citrate uptakes are similar in the inflamed paw (ip) and decreased steadily from Day 1 to Day 7. Lower accumulation in healthy tissues was obtained with [^68^Ga]Ga-4HMSA. Legend: blader (bl), injected paw (ip), heart (he) liver(li), kidneys (ki)

PET/CT images of mice injected with CFA displayed an accumulation of both tracers at the site of inflammation in the paw (Fig. 7). Comparison of the two sets of figures clearly shows that uptakes in healthy organs are significantly reduced for [^68^Ga]Ga-4HMSA, which was cleared relatively rapidly from blood circulation by the kidneys and bladder. By contrast, there is an accumulation of [^68^Ga]Ga-citrate in the upper body of the animal, such as the liver, lungs and the heart (Fig. 7B).

**Fig. 8:**
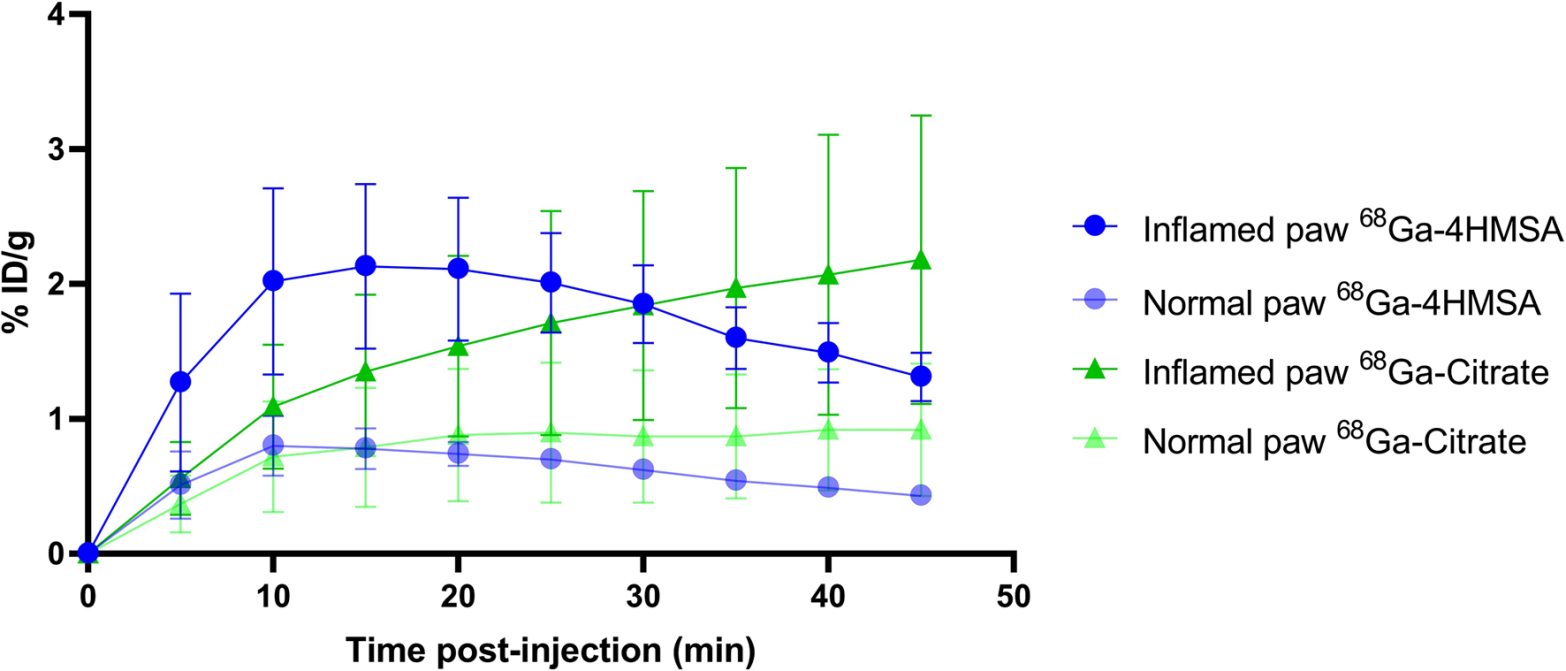
Paws uptake of [^68^Ga]Ga-4HMSA and [^68^Ga]Ga-citrate at Day 1 post-inflammation

To quantify the radiotracer uptake in the inflamed paw, regions of interest (ROI) were drawn on the inflamed paw using the AMIDE software [16] and the contralateral (non-injected) paw, which was used as a control. These ROI were then applied to all frames to obtain time activity curves (TAC) for the inflamed and normal paws. The ROI activity was expressed as percentage of injected dose per gram of tissue (%ID/g). It is worth noting that [^68^Ga]Ga-4HMSA activity grows rapidly in the inflamed paw to reach a maximum at 15 min and drops asymptotically to less than 10% of the maximum after 45 min, while that in the non-injected paw remains low (Fig. 8). This is in contrast to [^68^Ga]Ga-citrate activity, which grows steadily in the inflamed paw with the maximum value at 45 min, without significant apparent accumulation in the contralateral control paw. For the comparative studies, we selected the %ID/g value in the inflamed paws at the optimal uptake times for the [^68^Ga]Ga-4HMSA and [^68^Ga]Ga-citrate, which are at 15 min and 45 min, respectively.

[^68^Ga]Ga-4HMSA showed an uptake in the inflamed paw that was optimal at day 1 (2.13 ± 0.61), which then slowly decreased over time to reach 1.77 ± 0.61; 1.07 ± 0.26 and 1.49 ± 0.54%DI/g for days 2, 3 and 7 respectively (Table 5). These results are in accordance with the degree of inflammation in the foot pad following a subcutaneous injection of complete Freund’s adjuvant, which induces a prolonged swelling that becomes maximal at 24 h and persists for at least 7 days [20]. When we compare the %ID/g value in the inflamed paws at their optimal uptake times, both radiotracers showed very similar uptakes over time. The activities found in the contralateral (control) paws were statistically inferior to that of inflamed paws at days 1, 2 and 3 for both [^68^Ga]Ga-4HMSA and [^68^Ga]Ga-citrate (Table 5).

**Table 5:** PET uptake of [^68^Ga]Ga-4HMSA and[^68^Ga]Ga-citrate in BALB/c mice injected with CFA in the footpad at different time point post-CFA administration

Time post-CFA administration	[^68^Ga]Ga-4HMSA (%ID/g)		[^68^Ga]Ga-citrate (%ID/g)	
(Day)	Inflamed paw	Normal paw	Inflamed paw	Normal paw
1	2.13 ± 0.61**	0.78 ± 0.15	2.18 ± 1.07*	0.92 ± 0.49
2	1.77 ± 0.61*	0.91 ± 0.23	1.54 ± 0.48*	0.76 ± 0.08
3	1.07 ± 0.26**	0.69 ± 0.18	1.01 ± 0.29**	0.45 ± 0.12
7	1.49 ± 0.54	0.54 ± 0.10	1.40 ± 0.62	0.50 ± 0.10

Two-tailed independent Student’s *t-test*. non-significant: *p* > 0.05; *: *p* ≤ 0.05; **: *p* ≤ 0.01

**Fig. 9:**
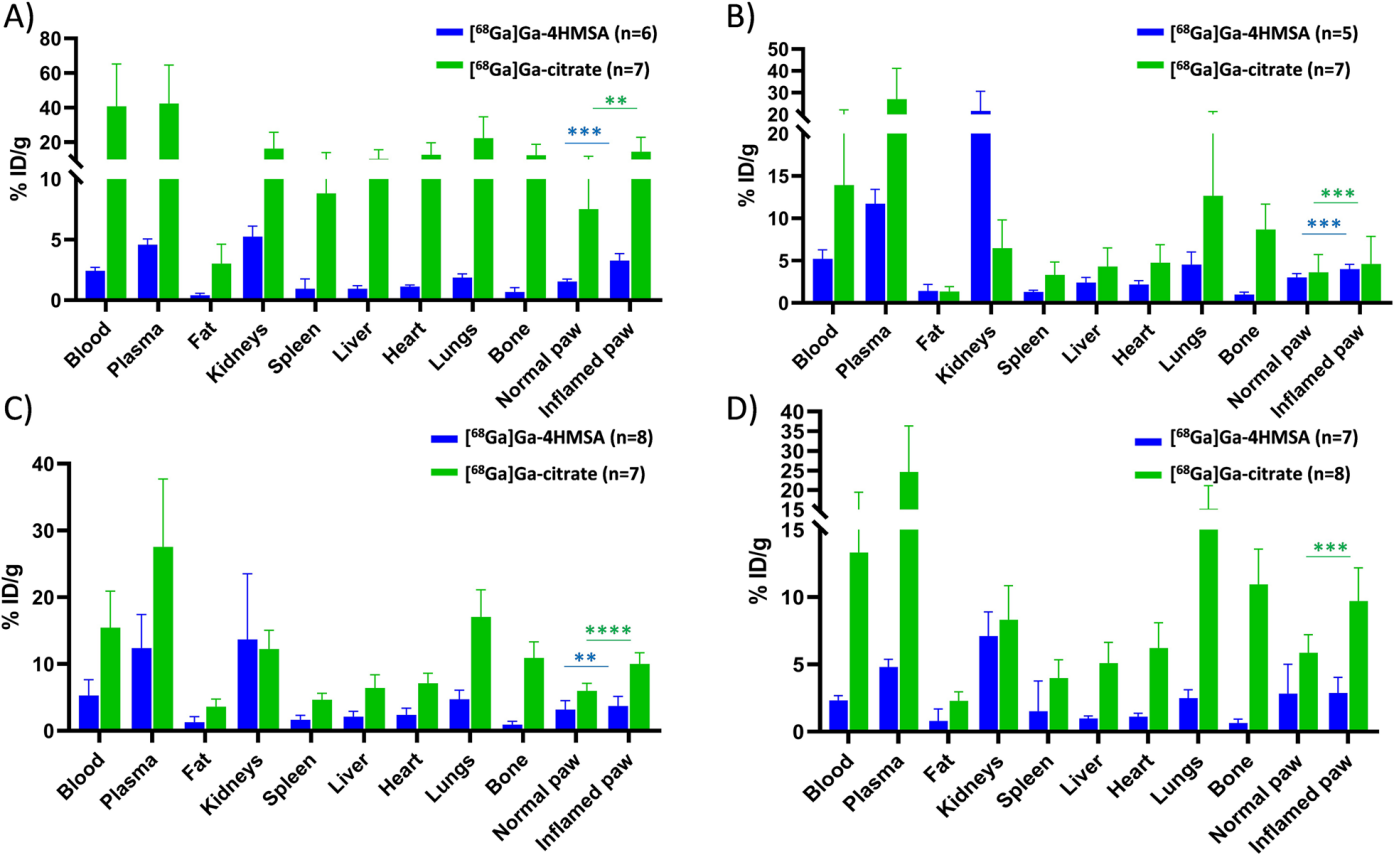
Biodistribution of [^68^Ga]Ga-4HMSA and [^68^Ga]Ga-citrate in BALB/c mice injected with CFA in the footpad at days 1 (**A**), 2 (**B**), 3(**C**) and 7 (**D**) post-inflammation. Biodistribution was performed 60 min after radiotracer injection. **p ≤ 0.01; ***p ≤ 0.001 ****p ≤ 0.0001

Biodistribution studies were performed in CFA-injected mice with either [^68^Ga]Ga-4HMSA or [^68^Ga]Ga-citrate. The animals were euthanized at 1-, 2-, 3-, or 7-days post inflammation and 1 h post radiotracer injection. Blood and various organs were removed to measure tissue-specific activity. For [^68^Ga]Ga-4HMSA the radioactivity was mostly concentrated in kidneys (5.26 ± 0.86, 11.94 ± 5.40, 16.06 ± 7.65 and 12.86 ± 4.96%ID/g at days 1, 2, 3 and 7, respectively) indicating renal elimination. At day 3 post-inflammation, higher retention of radioactivity was seen for [^68^Ga]Ga-4HMSA in almost all organs, blood and plasma as compared to days 1, 2 and 7 (Fig. 9). By contrast, accumulation of the [^68^Ga]Ga-citrate in various organs remained the same at different time point post-inflammation. Overall, the biodistribution profile of [^68^Ga]Ga-4HMSA in mice revealed lower uptake in the majority of the organs compared to [^68^Ga]Ga-citrate (Fig. 9). The higher retention of radioactivity in the lungs, the heart and in the bones observed in PET images of [^68^Ga]Ga-citrate is in accordance with the literature [22, 23].

The activities in the inflamed paws were statistically superior to that of the contralateral paws at days 1 to 3 for [^68^Ga]Ga-4HMSA and at all time points for [^68^Ga]Ga-citrate following biodistribution (Table 5). Surprisingly, the inflamed paw uptake values obtained from biodistribution for [^68^Ga]Ga-citrate (15.81 ± 6.33, 7.37 ± 1.21, 9.73 ± 1.59 and 9.33 ± 2.38%ID/g for 1-, 2-, 3- and 7-days post-inflammation, respectively) are higher than those of [^68^Ga]Ga-4HMSA (3.27 ± 0.58, 2.13 ± 0.19, 5.89 ± 2.52 and 5.24 ± 2.86%ID/g at day 1, 2, 3 and 7 post-inflammation, respectively (Fig. 9) and the ones obtained both from PET imaging (Table 5). We should mention that during the dissection of the paw, both bone and inflamed tissue were collected. A potential explanation for the discrepancy between biodistribution and imaging results could be explained by the high bone uptake that contributes to increase normal and inflamed paw uptakes obtained from biodistribution. It is worth noting that both radiotracers gave very similar inflamed to normal paw ratios from biodistribution and PET imaging over time (Table 6).

**Table 6:** Inflamed to normal paw ratios of [^68^Ga]Ga-4HMSA and [^68^Ga]Ga-citrate from PET imaging and biodistribution results

Time post-CFA administration	Inflamed/normal paw ratios from PET imaging		Inflamed to normal paw ratios from biodistribution	
	[^68^Ga]Ga-4HMSA	[^68^Ga]Ga-citrate	[^68^Ga]Ga-4HMSA	[^68^Ga]Ga-citrate
Day 1	2.73 ± 0.59	2.54 ± 0.67	2.13 ± 0.28	1.94 ± 0.35
Day 2	1.94 ± 0.18	2.03 ± 0.62	1.32 ± 0.06	1.71 ± 0.23
Day 3	1.56 ± 0.15	2.27 ± 0.61	1.19 ± 0.07	1.69 ± 0.23
Day 7	2.72 ± 0.65	2.79 ± 0.96	1.34 ± 0.68	1.67 ± 0.30

## Discussion

With the commercialization of ^68^Ge/^68^Ga generators and the high ^68^Ga cyclotron production capacity [9–11], the development of ^68^Ga-based radiotracers has increased considerably in the last few years for PET imaging. The promising results obtained with the new class of chelators we developed [12] led us to design an acyclic chelator 4HMSA for ^68^Ga complexation and imaging of inflammation by PET.

4HMSA was synthesized over seven steps with a high overall yield of 75%. This acyclic chelator offers a very fast labeling kinetic at room temperature with ^68^Ga under various pH (1.0–3.8) and an excellent AMA that is 8 times higher compared to DOTA analog. [^68^Ga]Ga-4HMSA demonstrated a remarkable resistance to transchelation and transmetalation by remaining almost unchanged when incubated with large excess of DTPA and biologically relevant metal ions *except with Fe*^*3*+^ (Tables 2 and 3). [^68^Ga]Ga-4HMSA remained also intact when incubated 2 h in human plasma and 1 h in mouse (Fig. 6) suggesting substantial in vitro and in vivo inertness in circulation.

The data obtained at day 3 after CFA inflammation show an increase in the uptake values of [^68^Ga]Ga-4HMSA at the majority of the organs, blood and plasma as compared to days 1, 2 and 7 (Fig. 9). At this time, there is no clear explanation for the increased activity of [^68^Ga]Ga-4HMSA on various tissues at day 3. While the activity found in the inflamed paws did not increase at this time point, its % ID/g value is statistically superior to that of contralateral paw (*p* ≤ 0.01) (Table 5). Overall, the results support the potential of [^68^Ga]Ga-4HMSA to target the inflammation region with a suitable pharmacokinetics for imaging, i.e. fast clearance from organs and circulation with renal excretion as the major route of elimination. It seems that 4HMSA acts as a transporter of ^68^Ga by modulating favorably its biodistribution profile.

The higher inflamed and normal paw uptake for [^68^Ga]Ga-citrate obtained from biodistribution can be explained by the high bone uptake of this tracer. Even though the paw uptakes obtained from biodistribution for [^68^Ga]Ga-citrate are higher than that of [^68^Ga]Ga-4HMSA (Fig. 9), inflamed to normal paw ratios from biodistribution are similar over time for the two tracers (Table 6). Although not significant, the inflamed to normal paw ratios obtained from PET imaging are superior compared to those from biodistribution. A potential explanation is that the biodistributions were done 1 h post-injection and this is not the optimal time selected for [^68^Ga]Ga-4HMSA (15 min) and [^68^Ga]Ga-citrate (45 min) from dynamic PET imaging studies. [^68^Ga]Ga-citrate in mice is slowly eliminated and has a high blood pool activity [17], which are in accordance with our results (Fig. 9).

[^68^Ga]Ga-citrate was chosen as a comparative radiotracer since it was successfully used as a control in the past for PET imaging in an inflammatory pain mouse model [23]. [^68^Ga]Ga-citrate is not stable in vivo. Once injected, the [^68^Ga]Ga-citrate is quickly dissociated releasing ^68^Ga^3+^ into the bloodstream since citrate is a weak *in-vivo* chelator. Then, 99% of the ^68^Ga^3+^ are bound to transferrin and other iron-binding proteins such as lactoferrin, ferritin [24, 25], which accumulate in inflammatory lesions. Indeed, at least two mechanisms have been proposed to explain the presence of ^67/68^Ga in the site of inflammation: 1) leukocyte labeling and 2) lactoferrin binding by high availability of ^67/68^Ga at the site of inflammation that could be explained by an increase of vessel permeability [26].

Although the increased vessel permeability can also be responsible of the higher amount of [^68^Ga]Ga-4HMSA at the site of inflammation, a very low percentage of this tracer (~ 10%) bound to plasma protein in blood circulation and the mechanism of action of [^68^Ga]Ga-4HMSA might be slightly different from that of [^68^Ga]Ga-citrate. However, a direct transchelation of ^68^Ga from [^68^Ga]Ga-4HMSA to lactoferrin [27] that is upregulated at the site of inflammation [28] could be possible.

In CFA-induced chronic inflammatory pain model, lipocalin 2 (Lcn2 also known as siderocalin or neutrophil gelatinase-associated lipcalin, NGAL) expression was strongly induced in the inflamed paws, peaking at 12 h after CFA injection and then gradually dropping [29]. [^68^Ga]Ga-4HMSA uptakes in the inflamed paw follow the same trend. Lcn2 are expressed by macrophages, neutrophils and various epithelial cells and are up-regulated in response to inflammatory stimuli [30]. It has been reported that Lcn2 can sequester enterobactin and also different catecholates, but unable to sequester DFO [30]. 4HMSA is structurally different than DFO and might be recognized by Lcn2. All these results open a window for another potential molecular mechanism, which might contribute to explain the presence of [^68^Ga]Ga-4HMSA at the site of inflammation. Possibly [^68^Ga]Ga-4HMSA can be sequestered by Lcn2 that are upregulated in the inflamed paws.

It should be noted that [^68^Ga]Ga-4HMSA was first evaluated in an animal model of sterile inflammation. Potential applications of this tracer would be imaging of sterile inflammatory diseases such as atherosclerotic, valvular, skin, bone and myocardial inflammation, vasculitis and arthritis [5]. The next valuable steps will be to investigate this promising PET tracer in models of infective inflammation.

## Conclusion

Our results confirm that both [^68^Ga]Ga-4HMSA and [^68^Ga]Ga-citrate are comparable radiotracers for the detection of inflammation. As a novel inflammation imaging agent, [^68^Ga]Ga-4HMSA showed high in vitro and in vivo stabilities, fast clearance from organs and circulation with renal excretion, which offer more advantages regarding the dosimetry and lower overall background activity. Further studies are warranted for a better understanding of the mechanism of action of this new PET tracer.

## Abbreviations

AMA: Apparent molar activity
CFA: Complete Freund’s adjuvant
CH_2_Cl_2_: Dichloromethane
CT: Computed tomography
DFO: Desferoxamine
DMSO: Dimethyl sulfoxide
DOTA: 1,4,7,10-Tetraazacyclododecane-1,4,7,10-tetraacetic acid
DTPA: Diethylenetriaminepentaacetic acid
EDTA: Ethylenediaminetetraacetic acid
[^18^F]FDG: 2-Desoxy-2-[^18^F]-fluoro-d-glucose
Fe: Iron
FOXE: Ferrioxamine E
^67^Ga: Gallium-67
^68^Ga: Gallium-68
GaCl_3_: Gallium trichloride
γ: Gamma
^68^Ge: Germanium-68
HCl: Hydrochloric acid
4HMSA: *N*-Hydroxy-*N*-methyl succinamide-based chelator
HPLC: High performance liquid chromatography
%ID/g: Percent injected dose per gram of tissue
IFN-γ: Interferon gamma
IL: Interleukin
IR: Infrared
ITLC: Instant thin layer chromatography
ITLC-SG: ITLC-silica gel
LC/MS: Liquid chromatography/mass spectroscopy
Lcn2: Lipocalin-2
MBq: Megabecquerel
MeOH: Methanol
MHz: Megahertz
NaCl: Sodium chloride
NGAL: Neutrophil gelatinase-associated lipocalin
NMR: Nuclear magnetic resonance
NOTA: 1,4,7-Triazacyclononane-1,4,7-triacetic acid
PET: Positron emission tomography
RCY: Radiochemical yield
ROI: Region of interest
Rf: Retention factor
rpm: Revolutions per minute
RT: Room temperature
SPECT: Single-photon emission computed tomography
TAFC: Triacetylfusarinine C
TAC: Time activity curve
THF: Tetrahydrofuran
TNF-α: Tumor necrosis factor alpha
TFA: Trifluoroacetic acid
TLC: Thin layer chromatography
UPLC: Ultra-performance liquid chromatography
^89^Zr: Zirconium-89
